# In Vivo Non-Destructive Monitoring of *Capsicum Annuum* Seed Growth with Diverse NaCl Concentrations Using Optical Detection Technique

**DOI:** 10.3390/s17122887

**Published:** 2017-12-12

**Authors:** Naresh Kumar Ravichandran, Ruchire Eranga Wijesinghe, Seung-Yeol Lee, Muhammad Faizan Shirazi, Hee-Young Jung, Mansik Jeon, Jeehyun Kim

**Affiliations:** 1 School of Electronics Engineering, College of IT Engineering, Kyungpook National University, 80 Daehak-ro, Buk-gu, Daegu 41566, Korea; nareshr.9169@gmail.com (N.K.R.); eranga@knu.ac.kr (R.E.W.); faizan_shirazi@knu.ac.kr (M.F.S.); jeehk@knu.ac.kr (J.K.); 2 School of Applied Biosciences, Kyungpook National University, 80 Daehak-ro, Buk-gu, Daegu 41566, Korea; leesy1985@gmail.com

**Keywords:** SS-OCT, *Capsicum annuum*, germination, salt concentration

## Abstract

We demonstrate that optical coherence tomography (OCT) is a plausible optical tool for in vivo detection of plant seeds and its morphological changes during growth. To investigate the direct impact of salt stress on seed germination, the experiment was conducted using *Capsicum annuum* seeds that were treated with different molar concentrations of NaCl. To determine the optimal concentration for the seed growth, the seeds were monitored for nine consecutive days. In vivo two-dimensional OCT images of the treated seeds were obtained and compared with the images of seeds that were grown using sterile distilled water. The obtained results confirm the feasibility of using OCT for the proposed application. Normalized depth profile analysis was utilized to support the conclusions.

## 1. Introduction

Germination of seeds is a growth process during which a plant is contained within its seed. Studies on seed germination and their respective morphological changes plays a vital role in attaining quicker harvesting [1,2,3,4,5]. Germination can be affected by a variety of internal factors as well as by external factors, such as the soil temperature [6], water oxygen content availability, light and dark conditions, soil salinity [7], soil pH [8], and soil chemical composition [9]. Most of these external factors vary with the seed type. Soil salinity plays a major role in germination [7,10,11]. Only certain (appropriate) soil salinity will promote germination. An excess amount of salinity will result in the soil degradation or in the inhibition of the germination process in seeds [12]. Salt content in water varies greatly from region to region, and from rain water to sea water. For instance, rain water usually has NaCl concentrations of 0.003 g NaCl/L and sea water concentrations of up to 0.030 g NaCl/L. Thus, choosing the right soil salinity is a major concern for farmers to maximize crop yield [13,14]. Many previous research studies have determined appropriate soil salinity conditions that promote germination, for a variety of plant species [7]. Earlier studies have shown the effect of seed priming by different salt concentrations, their advantages and disadvantages depending on the concentrations [4,11]. However, there have been no studies on in vivo monitoring of seed growth or inhibition of germination for a range of soil salt concentrations. Analysis and/or estimation of the germination growth stage, aiming to determine the effect of the level of inhibition on seed germination, has been accomplished using various methods, such as sectioning of seeds and microscope-assisted inspection [15,16], histological methods [17], scanning electron microscopy (SEM) imaging [18], magnetic resonance imaging (MRI) [19,20], and X-radiography [21]. All of these methods are either destructive methods that preclude continuous monitoring of the same seed or are inferior resolution-wise compared with optical coherence tomography (OCT).

OCT is an in vivo, non-contact, non-destructive real-time imaging method that was first introduced in 1991 [22]. The working principle of the OCT imaging method is based on the interferometric technique that uses a low-coherence light source. Because OCT offers non-contact real-time imaging, this method has been profoundly used for diagnostics in medical imaging and research studies that require high-resolution imaging of samples with thicknesses on the order of a few millimeters. Because it provides high-resolution images that are on par with histological images, OCT has been widely used in many fields, such as ophthalmology [23], otorhinolaryngology [24,25,26], dermatology [27], blood flow measurements [23,28], defect inspection and thickness measurements in electronics devices such as light-emitting diodes (LEDs) [29], liquid crystal displays (LCDs) [30], composite structural analysis [31], and agriculture. In past research studies by various groups, the usefulness of OCT in agricultural studies has been shown to be prominent, for example, for monitoring of leaf diseases [32,33,34], and for the identification of defects and diseases in seeds and fruits [35,36]. 

The experimental conditions and parameters of this study were based on previously reported studies on effects of salt stress on seed germination [4,11]. In the published literature articles, they have investigated the effect of salt concentrations of up to 200 mM NaCl on *Capsicum annuum* seed priming. This study was carried out as a continuous monitoring process for 9 days. All the seed specimens were inspected throughout the experimental duration, and only the representative OCT images (acquired on Days 1, 3, 5, 7, and 9) are shown in the manuscript, which emphasize representative major morphological changes. By demonstrating this, we show a broader spectrum of OCT applications as a tool for in vivo growth analysis of primed seeds. Two-dimensional (2D) OCT images of seeds exhibiting morphological changes during the growth within the seeds are presented, and the obtained results are supported by averaged and normalized depth profile analysis.

## 2. Materials and Methods

### 2.1. The Preperation of Seed Specimens and Experimental Conditions

To assure homogeneity and viability of the samples used in the experiment, the *Capsicum annuum* plant seeds that were utilized for experiments were obtained from the Department of Microorganisms, Kyungpook National University, Daegu, South Korea. The experimental procedure was approved by the research ethics committee of Kyungpook National University. All the seed specimens were selected with an average weight of ~1.44 g. The seed samples were washed using distilled water and dried at room temperature (27 °C). The experiment lasted for 9 consecutive days (the seeds exhibited prominent germination by the end of the experiment). Overall, 60 *Capsicum annuum* seeds were used in our experiments. These 60 seeds were partitioned into 4 groups, with each group containing 15 seeds. Seed specimens were placed on Petri dishes with each base-surfaced with tissue paper, were used as container bases for germinating the seeds, and was filled with individual solutions of sterile distilled water (SDW), 0.1 M NaCl (molar concentration of sodium chloride), 0.2 M NaCl, 0.3 M NaCl, and 0.4 M NaCl. The solutions were filled to a level slightly above the halfway point of the Petri dishes to ensure complete immersion of the seed samples into their individual solutions. To maintain similar saline environment conditions and to avoid dehydration during the OCT imaging process, each seed specimen was taken out and kept in a Petri dish containing a saline solution similar to its origin. All Petri dishes were handled with extreme care so that the seeds were not subject to any damages during the experiment. An external mark was placed on the top surface of the exact center of seed specimens using a normal marker to indicate the region of interest. This helped in identifying and maintaining the region of interest throughout the experimental duration.

### 2.2. Specimen Preperation Forhistological Analysis

For histological analysis, the *Capsicum annuum* seeds that were primed using sterile distilled water (SDW) were utilized. The seeds used for histological analysis were primed with SDW for 1 day. The samples that were used for histology were fixed in 2% paraformaldehyde and 2.5% glutaraldehyde in a 0.05 M sodium cacodylate buffer solution, for 24 h. Then, the samples were washed and dehydrated using graded ethanol series of 30%, 50%, 70%, 80%, and 90% of absolute ethanol for ~30 min. Then, the dehydrated samples were embedded in a Spurr’s resin after being infiltrated with propylene oxide. The specimen was then polymerized in 100% Spurr’s resin for 24 h at 70 °C. Finally, the samples were sectioned using an ultra-microtome (MT-7000, RMC, Tucson, AZ, USA) with a 2–3 µm thickness. The sectioned samples were stained with a 2% methylene blue solution to be inspected using a light microscope (BX50, Olympus, Tokyo, Japan).

### 2.3. OCT System Setup

**Figure 1:**
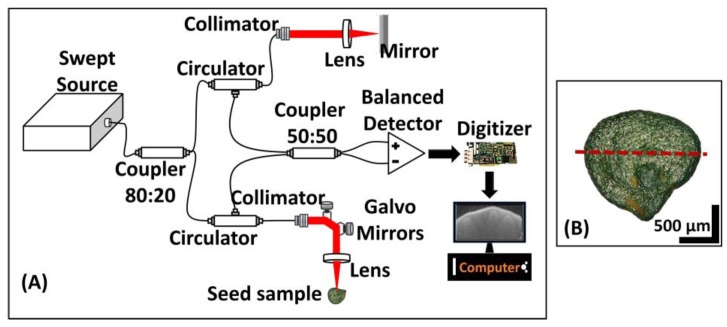
Schematic of the SS-OCT setup. (**A**) Schematic of the SS-OCT system. (**B**) Photograph of a sample *Capsicum annuum* seed. The dashed line represents the place of OCT scanning beam.

A custom assembled swept source optical coherence tomography (SS-OCT) was used for OCT imaging of the samples during the experiment. A schematic of the SS-OCT system that was used is shown in Figure 1A. The SS-OCT build was driven by a high-speed, broad bandwidth swept laser source (AXP50125-6, Axsun Technology, Billerica, MA, USA) with a center wavelength of 1310 nm and a full width at half maximum (FWHM) of 110 nm. The sweeping rate of the source was 50 kHz, with an average output power of 20 mW. The laser output from the source was connected to a fiber coupler (OCT-310C32C13, Gooch & Housego PLC, Ilminster, UK) at an 80:20 ratio. Twenty percent of the output arm of the coupler was then connected to the input arm of a circulator (CIR-1310-50-APC, Thorlabs Inc., Newton, NJ, USA), and the output arm of the circulator was connected to a reference arm setup. The reference arm setup consisted of a collimator followed by a focusing lens and a mirror. Similarly, the remaining 80% output from the coupler arm was also connected to the input arm of another circulator. The output arm of that circulator was connected to a sample arm setup. The sample arm consisted of a collimator, a galvanometer scanner, and a focusing lens. The final laser power at the sample arm end was 14 mW. The backscattered light beams from both the sample and reference mirrors were collected from their respective redirected circulator arms and connected to the input arms of a fiber coupler at a 50:50 ratio (OCT-310K32C13, Gooch & Housego PLC, Ilminster, UK). At this stage, the interference of the backscattered light beams from the reference arm and the sample arm occurred. The interference signal was then collected from the output arms of the 50:50 ratio coupler and connected to the positive and negative ports of a balanced photodetector (PDB430C, Thorlabs Inc., Newton, NJ, USA). All of the collimators, lenses, mirrors, and a galvanometer scanner (GVS102, Thorlabs Inc., Newton, NJ, USA) that were utilized in the system configuration were chosen so as to be optimal for working in the broad bandwidth range of wavelengths that was centered at 1310 nm. The signal obtained from the photodetector was digitized using a digitizer (ATS9462, Alazar Technologies Inc., Montreal, QC, Canada). 

The axial and lateral resolutions in air for the built system were 6.8 µm and 14.6 µm, respectively. The depth roll-off of the built system was less than 5 dB/mm, and the SNR of the system was 110 dB. To match the optical depth with the true depth of the sample, the obtained signals were multiplied with a factor of 1.42 (refractive index of seed structures). There are other methods to further improve the sensitivity fall-off at increasing depth [37,38]. A software-based data-processing technique was designed to construct the 2D OCT images. During the entire experimental duration, OCT imaging was performed in a controlled environment, in which the temperature was maintained at 27 ± 3 °C, and the humidity was in the 75–80% range, in a dark room. OCT imaging of the seed was maintained at the center of the seed. The region of interest (ROI) was determined by using a previously published article [6] as a base reference for identifying the important internal structures, and accordingly the ROI was selected. An external mark was placed on the ROI of seed specimens to indicate the position of OCT scanning, and multiple 2D-OCT scans were acquired approximately close to the aforementioned marked position, which made it possible to match the 2D-OCT image at the exact same imaging position as that of the images obtained on earlier days. Within the ROI, the cross-sectional image providing the maximum depth was selected for further analysis. The acquisition of multiple 2D-OCT images minimized the requirement of volumetric analysis, since the acquired cross sections sufficiently confirmed the desired results. Furthermore, the focusing point during scanning was set to be the same. To maintain similar saline environment conditions and to avoid dehydration during the OCT imaging process, each seed specimen was taken out and kept in a Petri dish containing the saline solution similar to its origin. All Petri dishes were handled with extreme care so that the seeds were not subject to any damages during the experiment. 

The seeds were placed (expected radical emerging point) facing the OCT scanning sample arm. Figure 1B shows the direction and position in which the seeds were placed during OCT imaging. Figure 1B shows the photograph of a representative *Capsicum annuum* seed and its imaging orientation, for which OCT imaging was maintained at the center of the seed, and the expected radicle emergence point was forward-facing during imaging. The red dashed line in Figure 1B indicates the direction and position of scanning by the sample arm. The total depth of the seed structures was not enough for the presented study to be carried out without any error occurrence due to intensity variations.

### 2.4. Depth Profile Analysis Algorithm

To analyze the obtained 2D images, a MATLAB (Mathworks, Middlesex County, NJ, USA) based software program was used to detect the intensity peaks in the depth direction. During the depth profile analysis process, 2D OCT image was loaded and a peak search algorithm based image window with 60 intensity signals (depth profile signals) was applied to select the region of interest. The peak search algorithm detects the maximum intensity in an individual depth scan sequentially. Due to the physical structure of the leaf, the 2D OCT cross-sectional image is an unflattened image containing maximum intensity index positions at different positions. All peaks positions in all 60 depth scans were rearranged to match the peak intensity index in the depth scans to flatten the image. Averaging these consecutive depths scans within a certain selected window aided in reducing the noise in the depth profile plots. The obtained depth profile intensities were then divided by the maximal value to obtain normalized depth profile intensity plots for the 2D OCT images. Thus, the absence of intensity owing to the presence of the air region can be considered negligible and did not negatively affect the plotted depth profiles.

## 3. Results and Discussion

**Figure 2:**
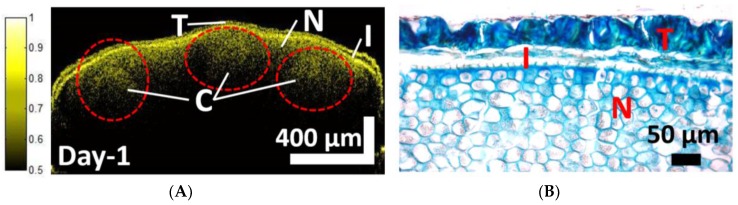
A 2D SS-OCT image and a histological image. (**A**) A 2D-SSOCT image of a seed primed with sterile distilled water for one day. (**B**) An enlarged histology image of the same seed that was used in (**A**). C: Cotyledon; I: Inner seed coat; N: Non-micropylar endosperm; T: Testa. Dashed red circle shows the enclosed cotyledon regions.

A 2D SS-OCT image and a histologically sectioned image of *Capsicum annuum* seeds, both of which were acquired during the initial SDW priming stage (Day 1), are shown in Figure 2. Figure 2A shows a 2D SS-OCT image of the primed seed, and Figure 2B shows an enlarged histology sectioned image of the same seed. It usually becomes difficult to distinguish between the cotyledon and the non-micropylar endosperm layers. However, the OCT image is based on change in the refractive index of the scattering layers in the sample, and different biological tissues have their respective refractive index coefficients. In seeds, the biological compositions of cotyledon and non-micropylar endosperm are different, which results in a difference in their intensity in OCT images. In Figure 2A, the red color dashed circles depict the corresponding borders of the cotyledon layers and the layer with comparatively lesser intensity; i.e., the non-micropylar endosperm layer around the cotyledon can be differentiated and identified with the help of a histology image. From comparative analysis, it can be concluded that the 2D OCT image reveals a similar growth pattern of seed structures in the testa, inner seed coat, and cotyledon. As per seed growth, the cotyledon and the embryo growth can be observed in 2D OCT images, which are shown later on.

**Figure 3:**
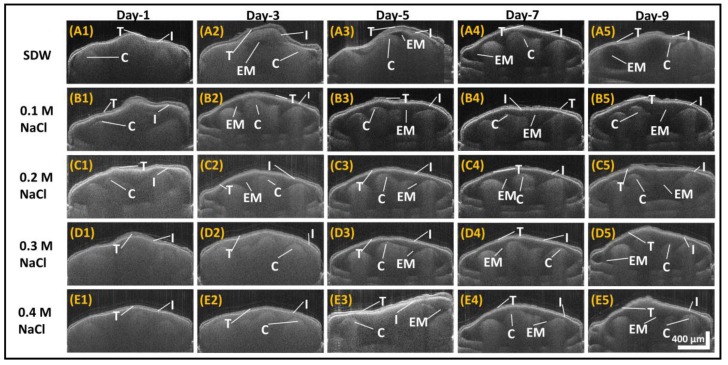
Comparative growth analysis by 2D SS-OCT of *Capsicum annuum* seeds primed with different salt solutions. The images were taken on consecutive days for seeds that were soaked in sterile distilled water (SDW) (**A1**–**A5**), 0.1 M NaCl solution (**B1**–**B5**), 0.2 M NaCl solution (**C1**–**C5**), 0.3 M NaCl solution (**D1**–**D5**), and 0.4 M NaCl solution (**E1**–**E5**). C: Cotyledon; EM: embryo; I: Inner seed coat; T: Testa. The scale bar of 400 µm applies to all 2D images.

**Figure 4:**
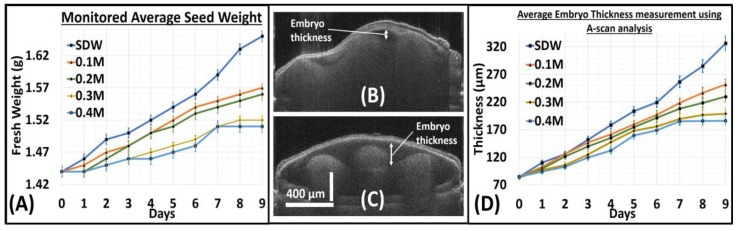
Statistical analysis of 2D OCT images using depth profile analysis. (**A**) The recorded average seed weight fluctuations during monitoring period. (**B**,**C**) Representative 2D-OCT images marked with embryo region in a seed. (**D**) The measured average embryo thickness using depth profile analysis of all seed groups during the monitoring period.

Figure 3 shows the SS-OCT images that were taken on consecutive days, for seeds that were soaked in SDW, 0.1 M NaCl, 0.2 M NaCl, 0.3 M NaCl, and 0.4 M NaCl solutions. Figure 3 shows the representative images of each corresponding seed category and provides visualizations of the inner morphological structures. The best seed image, with a visible structural difference in each group, was selected and monitored, and the images of that particular seed are arranged in Figure 3. The OCT in vivo imaging was carried out for all the seeds in each group. The statistical values giving the seed growth has been shown in Figure 4 plots. Table S1 provides the measured seed weight (average, standard deviation, minimum and maximum values) and embryo thickness measurement (average, standard deviation, minimum and maximum values) using depth profile analysis for the entire group throughout the monitoring process. Panels (A1) to (A5) in Figure 3 show the images of seeds soaked in SDW that were taken on consecutive days. It can be clearly seen that on Day 1 the three major distinct layers of seeds are visible: the testa (seed coating), the cotyledon, and the inner seed coat. Starting from Day 3, notable traces of collective vacuum-like deposition begin to appear beneath the inner seed coat layer, owing to the development of embryos. As the seed grows, the embryo becomes clearer and the cotyledon layer forms into multiple lobe-like structures, which is observable in images taken on Day 5 and Day 7. The bottom part of the seed starts to exhibit more structural details as the seed germination increases. This is visible on Day 7 onwards. On Day 9, the embryo is seen in most parts of the seed; in addition, the cotyledon appears to have a smaller area than the area observed during the preceding seed growth days. On Day 9, the seeds are apparently well-germinated.

In Figure 3, Panels (B1) to (B5) show the images of the seeds primed with the 0.1 M NaCl salt solution. On Day 1, the effect of the 0.1 M NaCl solution on the structural development of the seeds appears to be similar to that of the SDW solution. The development of the embryos and bottom structures of the seeds becomes barely visible from Day 3 onwards. The notable growth of the embryos and cotyledon structures becomes clearer as the seeds grow further. However, the difference between the states of the seeds on Day 7 and Day 9 appears to be insignificant. In addition, on Day 9 the embryos are not as prominent as those of the seeds grown in SDW.

Panels (C1) to (C5) in Figure 3 show morphological changes that occur internally in seeds soaked in the 0.2 M NaCl solution. Unlike the Day 1 images of SDW-primed seeds and 0.1 M NaCl primed seeds, the Day 1 images of 0.2 M NaCl seeds reveal a barely visible bottom structure in the imaged seeds. These structures, along with the embryo growth, appear at Day 3. The images of the seeds soaked in the 0.2 M NaCl solution, acquired on Day 3 and Day 5, reveal that the seeds exhibit fairly similar changes in their internal morphological structures compared to the Day 3 and Day 5 images of the seeds soaked in the 0.1 M NaCl solution. The intensities of structures in the Day 7 and Day 9 images of the seeds soaked in the 0.2 M NaCl solution are slightly higher than those in the Day 7 and Day 9 images of the seeds soaked in the 0.1 M NaCl and SDW solutions. This can seemingly be the effect of a slower germination rate induced by the 0.2 M NaCl solution.

Panels (D1) to (D5) in Figure 3 show the images of the *Capsicum annuum* seeds primed with the 0.3 M NaCl solution. It can be noted that, unlike on Day 1 and Day 3 for SDW-soaked seeds and those soaked in the 0.1 M NaCl and 0.2 M NaCl solutions, the embryos are not prominently visible on Day 1 and Day 3 for the 0.3 M NaCl primed seeds. The development of the embryos in these seeds appears to start on Day 5. In addition, the Day 7 image is quite similar to the Day 5 image. In the Day 7 and Day 9 images, the bottom part of the seed structures become visible, while in the case of SDW, 0.1 M NaCl, and 0.2 M NaCl primed seeds, these structures become visible on earlier days. In addition, the intensity of these structures is seemingly higher than that in other solutions.

Panels (E1) to (E5) in Figure 3 show the effect of the 0.4 M NaCl concentration on the growth of *Capsicum annum* seeds. It can be observed that the development of embryos starts on Day 5 in the seeds primed with the 0.4 M NaCl solution, which correlates with the Day 5 onset of development for the seeds primed with the 0.3 M NaCl solution. However, the visibility of the bottom structures of the seeds in Day 7 and Day 9 images for the 0.4 M NaCl solution is even weaker, compared with those of the seeds primed with the 0.3 M NaCl solution. In addition, for the seeds primed with the 0.4 M NaCl solution, the imaged internal morphological changes on Day 9 were much weaker than those on Day 7. Even naked eye examinations of these seeds, compared with the seeds primed with the 0.3 M NaCl solution, revealed weaker germination.

In Figure 3, the difference in size of the horizontal scale and the vertical scale bar is due to the fact that the scan range and the imaging depth of the OCT system were not same. The horizontal scale bar is dependent on the scan range, whereas the vertical scale bar is dependent on the depth range that can be achieved using the OCT system. The variation of image background intensities in Figure 3 is due to the time-dependent continuous power fluctuations of the swept source laser. Since the imaging procedure was started with SDW primed seeds, the contrast of the entire first row appears to be different from that of the others. The effect of the unstable laser power was compensated by normalizing the overall intensity of the depth profiles. Since time-consuming histological analysis for each seed specimen was not possible for monitoring experiments, the cotyledon, embryo, and other internal layers of the seeds were pre-identified by correlating them with histological images. The unique structural differences as well as the similarities in the seed specimens can be observed in the images shown in Figure 3. Even though at first glance, B5 and C5 might look similar, upon closer inspection, the structure of the testa and the bottom right internal layers of B5 and C5 are uniquely different from each other. These were taken at the center point of both seeds, which may seem to be a reason for the similarity in their appearances. Furthermore, the difference in the appearance of E3 and E4 is due to the change in orientation and the angle of seed specimen, since it had to be slightly shifted during imaging to overcome the negative effects of excessive back-reflection. We selected these images because only these images optimally showed the internal structural differences.

Figure 4 shows the statistical analysis for all groups. Figure 4A shows the average seed weight (providing average and standard deviation for 5 groups 15 seeds) of all five groups for each day during monitoring. Each seed group had an average initial fresh weight of approximately 1.44 g on Day 0. Seeds primed in SDW had the highest weight gain at the end of the monitoring period. It is to be noted that seeds primed in 0.1 M NaCl and 0.2 M NaCl were almost similar in weight, where 0.1 M NaCl primed seeds were only a few grams higher than those primed in 0.2 M NaCl. The weight values of the seeds primed in 0.3 M NaCl and 0.4 M NaCl were well below the other groups. Furthermore, both of these seed groups had a negligible gain in weight towards the end of the monitoring period. The average weight gain observed in each seed groups, along with its standard deviation value and the maximum and minimum weight of the seeds in each group that was observed during the entire monitoring process, is given in the Supplementary Materials.

For further structural analysis of seed morphology changes, we utilized a single point depth profile analysis procedure. We optimized the MATLAB-based program (stated earlier in Section 2.2 in Materials and Methods) such that the program takes individual point depth profiles of the entire 2D image, from which a single depth profile is chosen. The selection of the depth profile was made, and the maximum embryo thickness was observed. In Figure 4B,C, the embryo region in a 2D OCT image is marked with a double headed arrow for representation. The embryo region growth is a direct indication of the germination progress in a seed. Thus, by measuring the maximum embryo thickness in a seed, the growth progress can be determined. Figure 4D is a representative graph indicating the average embryo thickness measurement obtained using this depth profile analysis procedure for all the groups throughout the monitoring period. The averaged embryo thickness values for each group with its respective standard deviation, along with the maximum and minimum thickness observed in a seed in every group that was measured throughout the monitoring process is shown in the Supplementary Material. As previously mentioned in the OCT system setup, the axial resolution of the OCT system is 6.8 μm in air, while in seed it is 4.8 μm. Therefore, there can be a least measurement error of 4.8 μm in the thickness measurement of the embryo. The variation in the embryo is also proportional to the weight of seed, so a standard deviation of ~16 μm was observed, as shown in the Supplementary Material.

**Figure 5:**
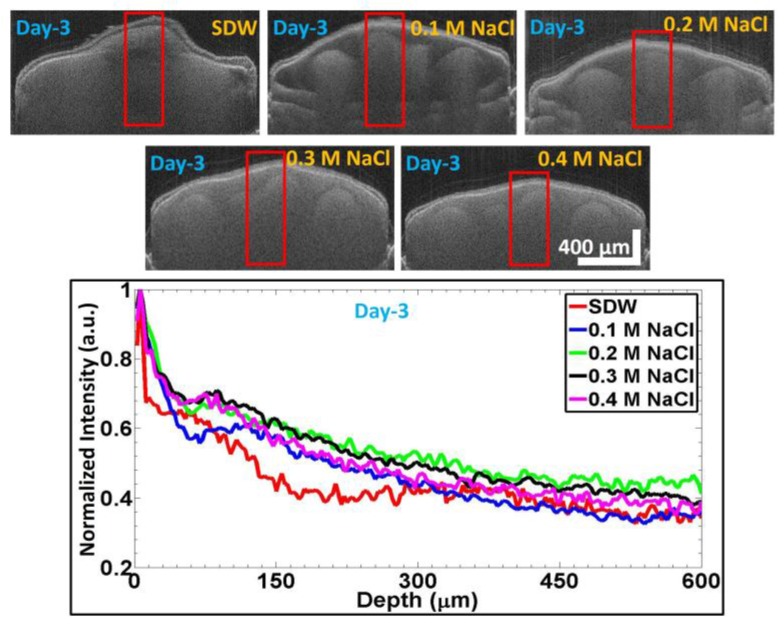
SS-OCT images acquired on Day 3 after priming the *Capsicum annuum* seeds with different solutions. The bottom figure is the respective averaged and normalized depth profile analysis plot (as discussed in Section 2.4) for SDW, 0.1 M NaCl, 0.2 M NaCl, 0.3 M NaCl, and 0.4 M NaCl, which are correspondingly shown with colors of red, blue, green, black, and magenta plots.

The top panel in Figure 5 shows the images acquired on Day 3, for seeds that were primed with different solutions. The bottom panel shows the normalized depth profile graphs for seeds in different priming scenarios. As the embryo development in a seed can be taken as a direct correlation for the germination progress, the ROI in the depth profile analysis was chosen in a way as to get the maximum development of the embryo region in the seed. Sixty consecutive depth profiles were taken within the window size (red rectangular box regions shown in the images in the top panel of Figure 5). The red curve corresponds to the normalized depth profile for the seeds that were primed with SDW, the blue curve is the normalized depth profile for the seeds primed with the 0.1 M NaCl solution, the green curve corresponds to the results for the seeds primed with the 0.2 M NaCl solution, the normalized depth profile plot for the seeds primed with the 0.3 M NaCl solution is given by the black curve, and the pink curve shows the results for the seeds primed with the 0.4 M NaCl solution.

The major illustration of the acquired depth profiles was the sudden decrease of the signal intensity due to the change in refractive index of the scattering medium, i.e., the internal structures. The sudden intensity-drop or weaker signal intensity was observed due to the changes of the refractive index of the germinated seed structures. By using this, we can determine the thickness of layers. Moreover, through a continuous monitoring process, it is possible to measure the growth progress internal structures. The intensity falloff in the plots illustrates the extent of growth in the analyzed seeds. Weaker intensity corresponds to higher growth, and vice versa. By comparison, it is observed that the seeds primed with SDW exhibit the highest growth on Day 3, followed by the seeds primed with the 0.1 M NaCl solution. The maximal intensity (or, in other words, the slowest growth) was found for the seeds primed with the 0.3 M NaCl and 0.4 M NaCl solutions.

**Figure 6:**
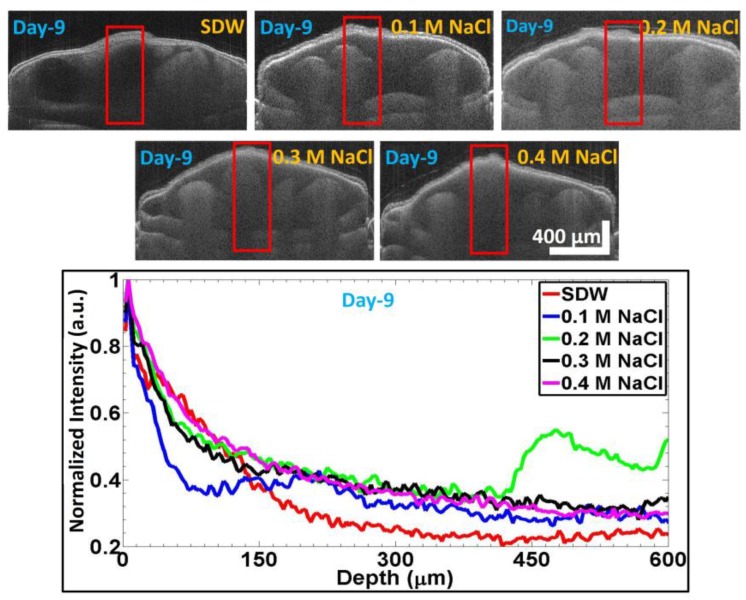
SS-OCT images on Day 9 after priming the *Capsicum annuum* seeds with different solutions. Bottom figure is the respective averaged and normalized depth profile analysis plot (as discussed in Section 2.4) for SDW, 0.1 M NaCl, 0.2 M NaCl, 0.3 M NaCl, and 0.4 M NaCl, which are correspondingly shown with colors of red, blue, green, black, and magenta plots.

Figure 6 shows the OCT images of seeds primed with the different solutions; the seeds were imaged on Day 9. The bottom panel shows the respective normalized depth profile plot for the seeds, acquired within the red rectangular boxes in the OCT images in the top panel. As mentioned above, these normalized depth profiles were plotted by averaging and normalizing 60 consecutive depth profile signals in each image. Owing to the maximal growth of embryos in SDW-primed seeds, the red curve shows the least signal intensity. The blue curve shows the second least intensity, showing changes in the seeds’ internal morphology. The results for the 0.2 M NaCl, 0.3 M NaCl, and 0.4 M NaCl primed seeds are shown with the green, black, and pink curves, respectively, and reveal much weaker growth than that in the other two solutions. The seed germination rates in SDW and NaCl solutions were evaluated by the internal seed regions, such as the embryos, the micropylar endosperm growth, and the cotyledons, which were formed by clearing the solid structures within the seed. A low germination rate was noted in seeds with higher NaCl concentrations. SDW-primed sample seeds yielded full germination as the seedling emerged, but negligible or no seedling emergence was noted for the seeds primed with the highest concentration of NaCl. This was confirmed with OCT images.

The biological nature, such as internal nutrients, illumination, and the refractive index, of the categorized 60 seed specimens (15 seeds × 4 groups), can be different from one to another. To minimize the aforementioned drawbacks, seed specimens with the exactly same weight were chosen, and such seeds were stored and imaged in a completely dark environment to maintain homogenous illumination and nutrient access. Therefore, the assumptions for the aforementioned seed biological nature were made on the basis of the weight. Moreover, the proposed study was carried out to examine how a feasible OCT imaging technique for in vivo germination progress assessment and for growth inhibition due to salt concentration. Future experiments aimed with other possible germination affecting factors can be studied for further evaluation of the system applicability.

## 4. Conclusions

We have demonstrated the usefulness of OCT as a tool for continuous in vivo monitoring of morphological changes in seeds. In addition, this study provided useful insights in the way OCT imaging can be used for seed growth analysis and for detecting the state of germination inhibition in seeds by continuous monitoring. Because maintenance of appropriate soil salinity plays a major role for seed growth, we conducted this study by priming *Capsicum annuum* seeds with different molar concentrations of salt and with sterile distilled water. We assessed the differences in internal morphological changes in seeds as the seeds grew. We observed that the seeds primed with sterile distilled water exhibited prominent growth, while the seeds primed with the 0.1 M NaCl and 0.2 M NaCl solutions exhibited slower but constant growth compared with the seeds soaked in sterile distilled water. Seeds primed with a higher concentration of NaCl showed negligible or no seedling emergence, indicating an impediment in the germination of the seed, which was verified with OCT monitoring images. The results of this study show the potential applications of OCT for agronomical studies related to seed growth. Further OCT studies of seed germination can help to determine appropriate conditions for faster seed germination, thereby reducing the time to harvest.

## Supplementary Materials

The following are available online at http://www.mdpi.com/1424-8220/17/12/2887/s1. Table S1, The average weight gain observed and the averaged embryo thickness values for each group, along with its standard deviation value and the maximum and minimum values of seeds in each group that was observed during the entire monitoring process.
