# Supplementary material for: In Vivo Non-Destructive Monitoring of Capsicum Annuum Seed Growth with Diverse NaCl Concentrations Using Optical Detection Technique
Source: Sensors (Basel). 2017 Dec 12;17(12):2887. doi: 10.3390/s17122887 (PMC5751711; doi:10.3390/s17122887)
Supplement: Supplementary file 1 [file sensors-17-02887-s001.pdf]

(A) Measured seed weight (g)

Days	SDW				0.1 M *NaCl*				0.2 M *NaCl*				0.3 M *NaCl*				0.4 M *NaCl*			
	Avg. (g)	StdDev.	Min. (g)	Max. (g)	Avg. (g)	StdDev.	Min. (g)	Max. (g)	Avg. (g)	StdDev.	Min. (g)	Max. (g)	Avg. (g)	StdDev.	Min. (g)	Max. (g)	Avg. (g)	StdDev.	Min. (g)	Max. (g)
0	1.44	0.00586	1.428	1.452	1.44	0.00584	1.428	1.452	1.44	0.00617	1.428	1.452	1.44	0.00718	1.426	1.453	1.44	0.00584	1.428	1.452
1	1.46	0.00688	1.446	1.474	1.45	0.00625	1.438	1.463	1.44	0.00679	1.43	1.454	1.44	0.00665	1.427	1.454	1.44	0.00616	1.448	1.452
2	1.49	0.00606	1.478	1.502	1.47	0.00602	1.458	1.482	1.46	0.00592	1.448	1.472	1.45	0.00577	1.438	1.462	1.45	0.00629	1.437	1.463
3	1.5	0.0066	1.487	1.513	1.48	0.00607	1.468	1.492	1.48	0.00624	1.468	1.492	1.46	0.00627	1.447	1.473	1.46	0.00685	1.446	1.473
4	1.52	0.0064	1.507	1.533	1.5	0.00588	1.488	1.512	1.5	0.00596	1.487	1.512	1.47	0.00667	1.457	1.483	1.46	0.00644	1.447	1.474
5	1.54	0.00596	1.528	1.552	1.52	0.00667	1.507	1.533	1.51	0.00726	1.495	1.525	1.48	0.00576	1.468	1.492	1.47	0.00651	1.457	1.483
6	1.56	0.00624	1.548	1.572	1.54	0.00587	1.528	1.552	1.53	0.00714	1.516	1.544	1.49	0.00715	1.476	1.504	1.48	0.00616	1.468	1.492
7	1.59	0.00695	1.576	1.604	1.55	0.00667	1.537	1.563	1.54	0.00616	1.528	1.552	1.51	0.00671	1.497	1.523	1.51	0.00638	1.496	1.523
8	1.63	0.00613	1.618	1.642	1.56	0.00703	1.546	1.574	1.55	0.00628	1.537	1.563	1.52	0.00619	1.508	1.532	1.51	0.00692	1.497	1.524
9	1.65	0.00683	1.636	1.664	1.57	0.00606	1.558	1.582	1.56	0.00673	1.547	1.573	1.52	0.0057	1.509	1.532	1.51	0.00658	1.497	1.524

(B) Embryo Thickness measurement using A-scan analysis

Days	SDW				0.1 M *NaCl*				0.2 M *NaCl*				0.3 M *NaCl*				0.4 M *NaCl*			
	Avg. (µm)	StdDev.	Min. (µm)	Max. (µm)	Avg. (µm)	StdDev.	Min. (µm)	Max. (µm)	Avg. (µm)	StdDev.	Min. (µm)	Max. (µm)	Avg. (µm)	StdDev.	Min. (µm)	Max. (µm)	Avg. (µm)	StdDev.	Min. (µm)	Max. (µm)
0	84.56	15.543	53.475	115.645	84.49	15.356	53.779	115.201	83.62	17.268	49.085	118.155	83.18	16.696	49.789	116.571	84.61	15.869	52.873	116.347
1	110.32	17.482	75.355	145.285	102.4	15.905	70.540	134.160	99.29	17.453	64.383	134.197	97.14	15.241	66.657	127.623	94.19	17.005	60.179	128.201
2	125.6	16.326	92.948	158.252	125.7	16.066	93.549	157.811	121.6	15.772	90.017	153.103	105.7	15.650	74.370	136.970	102.7	16.322	70.087	135.373
3	151.88	15.835	120.210	183.550	146.8	17.207	112.406	181.234	139.8	17.032	105.716	173.844	124.9	16.779	91.303	158.417	119.6	17.383	84.794	154.326
4	178.56	17.433	143.695	213.425	161.5	15.361	130.798	192.242	155.1	16.038	123.054	187.206	147.5	17.588	112.345	182.695	132.2	17.568	97.023	167.297
5	203.58	16.461	170.658	236.502	179.5	17.257	145.027	214.053	174.5	16.135	142.250	206.790	167.8	17.537	132.766	202.914	159.6	16.809	125.942	193.178
6	219.46	15.743	187.974	250.946	196.9	17.120	162.671	231.149	191.6	16.101	159.378	223.782	175.9	16.576	142.757	209.063	168.9	15.315	138.309	199.571
7	256.81	16.545	223.720	289.900	218.5	15.521	187.438	249.522	208.2	17.104	173.983	242.397	189.5	16.543	156.454	222.626	185.1	15.834	153.432	216.768
8	285.31	17.371	250.568	320.052	236.9	17.533	201.785	271.915	218.5	16.344	185.831	251.209	196.8	16.316	164.157	229.423	185.9	16.709	152.442	219.278
9	326.47	15.317	295.836	357.104	251.9	17.411	217.039	286.681	229.8	17.487	194.856	264.804	198.9	15.799	167.343	230.537	185.9	15.851	154.238	217.642
